# The Oral-Gut-Brain AXIS: The Influence of Microbes in Alzheimer’s Disease

**DOI:** 10.3389/fncel.2021.633735

**Published:** 2021-04-14

**Authors:** Narengaowa, Wei Kong, Fei Lan, Umer Farooq Awan, Hong Qing, Junjun Ni

**Affiliations:** ^1^Key Laboratory of Molecular Medicine and Biotherapy, School of Life Sciences, Beijing Institute of Technology, Beijing, China; ^2^Laboratory of Molecular Biology, Department of Botany, Government College University, Lahore, Pakistan

**Keywords:** oral-gut-brain axis, microbes, pathogens, neuroinflammation, Alzheimer’s disease

## Abstract

Alzheimer’s disease (AD) is one of the most frequently diagnosed neurodegenerative disorders worldwide and poses a major challenge for both affected individuals and their caregivers. AD is a progressive neurological disorder associated with high rates of brain atrophy. Despite its durable influence on human health, understanding AD has been complicated by its enigmatic and multifactorial nature. Neurofibrillary tangles and the deposition of amyloid-beta (Aβ) protein are typical pathological features and fundamental causes of cognitive impairment in AD patients. Dysbiosis of oral and gut microbiota has been reported to induce and accelerate the formation of Aβ plaques and neurofibrillary tangles. For instance, some oral microbes can spread to the brain through cranial nerves or cellular infections, which has been suggested to increase the risk of developing AD. Importantly, the interaction between intestinal microbiota and brain cells has been recognized as influencing the development of AD as well as other neurodegenerative diseases. In particular, the metabolites produced by certain intestinal microorganisms can affect the activity of microglia and further mediate neuroinflammation, which is a leading cause of neuronal necrosis and AD pathogenesis. Which pathogens and associated pathways are involved in the development and progression of AD remains to be elucidated; however, it is well-known that gut microbiota and their metabolites can affect the brain by both direct and indirect means. Understanding the specific mechanisms involved in the interaction between these pathogens and the nervous system is vital for the early intervention in AD. In this review, we aim to comprehensively discuss the possible mechanistic pathways underlying the oral-brain, the gut-brain and the oral-gut-brain associations.

## Introduction

First described by the German neuropathologist Alois Alzheimer in 1906, Alzheimer’s disease (AD) is a progressive neurodegenerative disorder characterized by continued cognitive and behavioral impairment such as the inability to make new memories and loss of primordial memories (Alzheimer, 1906; Goedert and Spillantini, 2006). Patients have difficulty reasoning and conceptualizing abstract ideas, and may even have problems with language (Willis et al., 1998), resulting in depression, difficulty sleeping, and increased anxiety. AD is a leading cause of mortality globally (World Health Statistics, 2020). Despite its debilitating effects, the specific etiology of AD remains unclear, and attempts at developing related treatment strategies have not obtained encouraging results.

In addition to the genetic risk factors that contribute to AD onset, several acquired factors, such as cerebrovascular disease, diabetes, hypertension, obesity, dyslipidemia, and microbial dysbiosis, also increase the risk of developing AD (Silva et al., 2019). The human body is colonized by trillions of different microorganisms, collectively known as the microbiota (Leser and Molbak, 2009). Microorganisms are accessory organs of the human body, and the microbial communities gathered in different parts of the body form a mutually beneficial symbiotic relationship with their host (Leser and Molbak, 2009). A recent study demonstrated that an increased abundance of pro-inflammatory *Escherichia Shigella* and a reduced abundance of anti-inflammatory *Eubacterium rectale* might be associated with systemic inflammation in AD patients (Cattaneo et al., 2017). Another study demonstrated that the fecal microbiota profile in AD patients was characterized by reduced microbial diversity, a decreased abundance of *Firmicutes and Bifidobacterium*, and an increased abundance of *Bacteroidetes* (Vogt et al., 2017). The gastrointestinal tract and the oral cavity are the main distribution sites of symbiotic microorganisms in the human body (Ogobuiro et al., 2020), and several studies have assessed the influence of oral microbiota on AD. A prospective observational study of patients with mild cognitive impairment (MCI) reported that those with periodontitis suffered a greater memory decline compared with MCI patients without periodontitis (Ide et al., 2016). Moreover, lipopolysaccharide (LPS) from *Porphyromonas gingivalis* (*P. gingivalis*), a bacterium commonly found in the oral tract, has been detected in the brains of AD patients (Poole et al., 2013), which raised a serious study focusing on the mechanistic relationship between periodontitis and AD (Ide et al., 2016; Wu et al., 2017; Ilievski et al., 2018; Dominy et al., 2019). Furthermore, a recent comparative study of postmortem cerebral tissue reported that, compared with aged people without AD, the brain of AD patients had a higher proportion of gingipains, the toxic protein produced by *P. gingivalis*, the keystone pathogen in chronic periodontitis. However, these gingipains were also present in the individuals not diagnosed with AD, albeit in smaller quantities, which was suggested to represent an asymptomatic preclinical stage of this disease (Dominy et al., 2019).

A large number of studies have shown that the oral microbial community and intestinal microbiota may be related to the occurrence and development of AD. This association may be direct (for instance, pathogenic microorganisms may directly invade the brain) (Poole et al., 2013; Dominy et al., 2019) or indirect (for instance, pathogenic microorganisms might trigger systemic inflammation that then leads to central nervous system [CNS] inflammation) (Liu et al., 2013). This review will comprehensively explore the role of the oral-gut-brain axis in the occurrence and development of AD.

## The Pathologies of AD

The main clinical hallmarks of AD patients include progressive memory loss, cognitive dysfunction, distracted attention, emotional disturbance, and personality changes. Meanwhile, the pathological features consist primarily of an excessive of amyloid-beta (Aβ) peptide in the cerebral cortex and hippocampus that leads to formation of senile plaques (SPs); neurofibrillary tangles (NFTs) in neuronal cells, and a reduction in the numbers of nerve cells in the cerebral cortex and hippocampus; The choline acetyltransferase (ChAT) and acetylcholine (Ach) content is also significantly reduced (Kenney et al., 2018). The predominant hypotheses for the underlying causes AD are the Aβ cascade hypothesis, the tau protein hyperphosphorylation hypothesis, and the oxidative stress and inflammation cascade hypothesis.

### The Aβ Cascade Hypothesis of AD

In general, the key factor for AD development is the excessive accumulation of Aβ protein in the brain. Aβ is a polypeptide fragment composed of 39–43 amino acid residues generated by the cleavage of amyloid precursor protein (APP, a 695-amino acid membrane-localized protein). In the brain, APP is sequentially cleaved by two enzymes, namely, beta-site APP cleaving enzyme (BACE) and gamma-secretase. Cleavage by gamma-secretase at different sites yields both Aβ_40_ and Aβ_42_ (Zhang et al., 2014).

Aβ protein can be engulfed by monocytes and neutrophils and degraded by Aβ degrading enzymes, or be phagocytosed by macrophages, or excreted through bile and urine. Under normal physiological conditions, a balance between Aβ production and clearance can prevent the excessive accumulation of the protein. When this balance is disturbed, Aβ protein will accumulate in the brain and form SPs, thereby inducing synaptic toxicity and neuronal death, damaging the blood-brain barrier (BBB), and aggravating the degeneration of the nervous system (Chen, 2015; Ferreira et al., 2015). The amyloid-beta hypothesis is the mainstream concept that has underpinned AD research for over 20 years; however, all attempts to develop Aβ-targeting drugs that can treat AD have failed. Consequently, other mechanisms have been postulated to be involved in AD pathology, in addition to Aβ accumulation.

### The Tau Hyperphosphorylation Hypothesis and AD

Tau is a microtubule-associated protein that regulates the stability of tubulin assemblies. It is mainly concentrated in axons, but is also present in dendrites (Carlyle et al., 2014). The tau hypothesis stipulates that hyperphosphorylation of the tau protein leads to the formation of NFTs, and ultimately to the onset of AD. Phosphorylated tau protein can bind to microtubules to maintain their stability. However, when hyperphosphorylated, tau will detach from tubulin and aggregate, eventually forming NFTs. This change is considered to be one of the causative factors of cognitive decline in AD patients (Qian et al., 2010). Data from both animal and human studies have suggested that neurons susceptible to tau pathology express signs of elevated cytosolic calcium; these vulnerable neurons also have increased calcium signaling during stress exposure. Hence, without the presence of regulatory factors, these neurons would be particularly vulnerable to tau pathology in the aging brain (Arnsten et al., 2020).

### Neuroinflammation and AD

Increasing evidence suggests that AD pathogenesis is not restricted to the neuronal compartment but is also strongly associated with immunological mechanisms in the brain, contributing to disease progression and severity (Heneka et al., 2018). Neuroinflammation is a leading cause of nerve cell necrosis, and it is also a trigger mechanism for AD. Neuroinflammation is mainly mediated by microglia, with perivascular myeloid cells and astrocytes playing an auxiliary role. The initial inflammatory response can activate microglia and astrocytes (Heneka et al., 2015; Businaro et al., 2018). Activated microglia release a variety of proinflammatory cytokines [including interleukin (IL)-1β, IL-6, and tumor necrosis factor (TNF-a)], chemokines, and reactive oxygen species (ROS), among others, which recruits more microglia and astrocytes to the site of inflammation. Microglia plays a role similar to that of macrophages in the immune defense of the CNS. Under normal circumstances, microglia that are recruited to the site of inflammation can remove irritants or pathogens, thereby protecting the brain from damage (Streit et al., 2014; Tang and Le, 2016). They can also internalize excess Aβ protein, preventing it from over-aggregating and causing SP-mediated damage to the CNS. Furthermore, studies have shown that continuous and progressive inflammation can lead to the accumulation of a large number of microglia and astrocytes at the site of inflammation, and the excessive release of TNF-α, IL-1β, IL-6 and other pro-inflammatory cytokines will further aggravate neuroinflammation and induce synaptic toxicity and neuronal death. Over recent years, an increasing number of studies have shown that inflammation, and especially the pleiotropic effects of microglia, may play a critical role in the development of AD (Heneka et al., 2015). Microglia cannot only remove stimuli and pathogens in the CNS, but can also affect synapse formation, synaptic plasticity, and neural stem cells (Minter et al., 2016). The differentiation and maturation of these cells in a proinflammatory environment may harm the brain and promote AD pathology.

## Oral Microbiota and AD

### Oral Microbiota

The oral microbiota occupies an extremely important position within the human microbiome, and is the second-largest microbiota in human after that in the gut (Fak et al., 2015). A suitable temperature, appropriate humidity, and abundant nutrition in the oral cavity provide a good living environment for microorganisms. Oral microbiota formation requires the initial attachment of bacteria to the host surface, typically to salivary molecules adsorbed to the surface. Once attached, the bacteria divide and secrete polymers that provide a matrix or scaffold for further microcolony development (Kolenbrander et al., 2006). Under normal conditions, biofilms maintain a homeostatic balance with their host; in disease, however, biofilms become dysbiotic (Lamont et al., 2018). The association between biofilms and a healthy gum is characterized by a limited commensal microbiota dominated by members of the phylum *Firmicutes*, including a diverse group of streptococcal species. Dental caries, an unusual disease, is known to involve cariogenic bacteria in dental plaques, such as acidogenic and acid-tolerant lactobacilli, and dietary fermentable carbohydrates that the bacteria convert to lactic acid (Gao et al., 2018). However, these bacteria are thought to be associated more with the progression rather than the initiation of the caries process (Van Houte, 1980). Periodontal diseases frequently occur in humans and can be divided into gingival disease and periodontitis (Agnello et al., 2017). Studies have suggested that species such as *P. gingivalis*, *Tannerella forsythia*, and *Treponema denticola*, known as the “red complex,” may be associated with periodontitis (Socransky and Haffajee, 2005). The oral microbiome represents a complex system that is markedly different in health and disease.

### Periodontitis and AD

Periodontitis has been shown to have a positive link with AD. A prospective observational study of patients with MCI reported that those with periodontitis suffered a greater memory decline over 6 months in comparison with MCI patients without periodontitis (Ide et al., 2016). *P. gingivalis* is the key pathogen in periodontitis, and LPS and gingipain from *P. gingivalis* have been detected in the brains of AD patients (Poole et al., 2013; Dominy et al., 2019), which raising a serious study focusing on the mechanistic relationship between periodontitis and AD (Ide et al., 2016; Wu et al., 2017; Ilievski et al., 2018; Dominy et al., 2019). The interaction among different oral microorganisms and between the oral microbiota and the host constitutes the oral micro-ecosystem. An imbalance in this micro-ecosystem may result in dental diseases such as caries, gingivitis, and periodontitis, as well as systemic diseases. In the occurrence of AD, it is thought that oral pathogenic microbes and their toxic substances damage the BBB and trigger or aggravate neuroinflammation, amyloid deposition and Tau protein phosphorylation, subsequently leading to cognitive impairment (Wu et al., 2017; Dominy et al., 2019; Zeng et al., 2020). The bacteria associated with AD are mainly periodontal pathogens, the plaques formed on the surface of teeth are mainly composed of Gram-negative bacteria, including *P. gingivalis*, *Aggregatibacter actinomycetemcomitans*, *Treponema denticola*, *Prevotella intermedia*, *Campylobacter rectus*, and *Tannerella forsythia* (Liccardo et al., 2020). In recent years, studies have shown that there may be a two-way effect between oral diseases and some systemic disorders (Jin et al., 2016). The oral microbiota may influence AD development through either direct or indirect routes.

### The Direct Effect of Oral Microbes on AD

In terms of direct effects, pathogenic oral microbes affect the occurrence of AD by entering the brain tissue through different paths and directly damaging the CNS (Parahitiyawa et al., 2009; Teixeira et al., 2017; Bulgart et al., 2020). Hemorrhagic oral treatment such as tooth extraction or transient bacteremia caused by gingivitis and periodontitis allows pathogenic microorganisms to break through the oral mucosal barrier and invade the bloodstream. The possible routes of bacterial entry into the bloodstream include the root canal, crossing from periapical lesions into alveolar blood vessels, or *via* gingival crevices to the capillaries in gingival connective tissues (Savarrio et al., 2005; Ewald et al., 2006; Nanci and Bosshardt, 2006). However, these microorganisms usually do not grow and multiply in the blood. They can only multiply and cause disease once the oral microorganisms enter the brain tissue through the bloodstream and cause damage to the nervous system (Geerts et al., 2002; Singhrao et al., 2015). Oral microorganisms can also enter the brain through the trigeminal nerve, which are connected to the brain (Teixeira et al., 2017). Microbes entering the brain tissue and the pro-inflammatory factors they released further trigger an immune cascade, which includes the activation of microglia, inflammatory responses, and the activation of the complement system (Bulgart et al., 2020). Studies have also suggested that Aβ protein, which functions as an antibacterial peptide in innate immunity, may inhibit neuroinflammation to a certain extent and play a protective role in the brain. However, oral microbes entering the CNS will trigger an immune response, thereby increasing the production of Aβ, and may even trigger the Aβ cascade to promote the occurrence of AD (Weaver, 2020).

Oral microorganisms that invade the bloodstream also need to break through the BBB to finally enter the brain tissue. The BBB, located between the periphery and the CNS, is composed of astrocytes, microglia and endothelial cells. Tight junctions between the cells limit the entry of pro-inflammatory factors, pathogens, or neurotoxic substances into the brain parenchyma. The permeability of the BBB is affected by numerous and complex factors, such as age, body temperature, and blood pH (Kiyatkin and Sharma, 2009). BBB permeability is increased in elderly AD patients, which facilitates the entry of oral microbes and their toxic factors into the brain tissue, thereby promoting the occurrence of AD. Additionally, some oral microorganisms and the cytokines and toxic substances they secrete may also alter the permeability of the BBB, making it easier for these microorganisms to penetrate the brain and cause damage to the CNS. Although Aβ is generated primarily by neurons in the brain, peripheral cells, including platelets, skeletal muscle cells, skin fibroblasts, and monocytes/macrophages, are also sources of Aβ production (Kuo et al., 2000; Evin et al., 2003; Roher and Kokjohn, 2009; Nie et al., 2019). We have previously reported that mice infected with *P. gingivalis* exhibit reduced BBB integrity, as evidenced by the lower numbers of tight junction-related proteins and the increased influx of Aβ from the periphery into the brain when compared with uninfected controls (Zeng et al., 2020). These observations suggest that oral microorganisms may enter into the brain through the impairment of BBB by themselves or by their toxic factors.

### The Indirect Effect of Oral Microbes on AD

Chronic oral diseases such as periodontitis can damage the oral mucosal barrier. This allows cytotoxins and pro-inflammatory factors released by oral microorganisms to travel through the bloodstream and enter the brain, where they activate inflammatory and immune responses, thereby indirectly affecting the process of AD. In addition, almost all chronic diseases leads to increased release of endothelin. Increased levels of endothelin-1 in the periodontal tissues of patients with periodontitis may affect the progression of AD (Scannapieco and Cantos, 2016). Furthermore, periodontitis, a chronic oral disease, may further cause systemic inflammation that triggers the microglia mediated innate immune response in the CNS (Liu et al., 2013). Periodontal disease can induce systemic inflammation in various ways, including through the direct action of toxic substances or their products or through the overexpression of cytokines and chemokines produced in periodontic lesions (Epstein et al., 1999). In addition, systemic inflammation can also be induced when oral pathogenic microorganisms migrate to the intestine and then to the circulatory system, or by the saliva secreted by the oral cavity may also cause systemic inflammation (Arimatsu et al., 2014). Interestingly, *P. gingivalis* can reportedly induce an increase in the expression of the pro-inflammatory cytokines TNF-α and IL-6 as well as promote non-alcoholic fatty liver disease, which, in turn, promotes neuroinflammation and causes neurodegenerative changes, which may trigger AD (Yoneda et al., 2012). We previously also reported that chronic systemic exposure to *P. gingivalis*-derived LPS (PgLPS) induces a systemic inflammatory bone loss-related cognitive decline in middle-aged mice through common risk factors of IL-6 and IL-17 (Gu et al., 2020). These observations suggest that the indirect effects of oral bacterial on the brain may be mediated by systemic inflammation, which induces cognitive impairment as a consequence of neuroinflammation.

## Intestinal Microbiota and the Pathogenesis of AD

### Intestinal Microbiota

Bacteria, fungi, and viruses constitute the intestinal microecosystem, with bacteria accounting for the greater proportion. The intestinal microbiota has been called the “second human genome” (Zhu et al., 2010). Compared with that in the oral cavity, the intestinal microbiota shows the greater diversity (Deo and Deshmukh, 2019). The number of these microorganisms can reach 1 × 10^12–14^ in the colon, making intestinal microbiota one of the most densely populated communities anywhere, far exceeding that of the soil, subsoil, and oceans (Icaza-Chavez, 2013). The diversity of the intestinal microbiota can be modulated by food products, dietary habits, and geographical origin (Senghor et al., 2018). The intestinal microbiota is predominantly composed of bacteria from three major phyla, namely, *Firmicutes*, *Bacteroidetes*, and *Actinobacteria* (Tap et al., 2009). Alterations in the composition of the intestinal microbiota caused by dietary changes, antibiotic exposure, and infections lead to imbalances in homeostasis, which may further promote the development of many diseases, such as colorectal cancer (Wang et al., 2012), obesity, inflammatory bowel disease (IBD) (Machiels et al., 2014), diabetes (Murri et al., 2013), heart failure (Geleijnse et al., 2004), and neurodegenerative disorders (Pistollato et al., 2016). Studies have shown that the intestinal microbiota is an important source of neurotransmitters (Strandwitz, 2018), including dopamine, noradrenaline, serotonin, gamma-aminobutyric acid (GABA), acetylcholine, and histamine (Stanaszek et al., 1977; Tsavkelova et al., 2000; Landete et al., 2007; Shishov et al., 2009; Pokusaeva et al., 2017), which suggests that changes in the intestinal microbiota may play a critical role in the development and progression of AD.

### The Direct Effect of Gut Microbes on AD

Small bacterial metabolites can translocate and diffuse systemically, or even pass through the BBB, and reach the cerebrospinal fluid (CSF) under healthy or specific conditions (Obrenovich, 2018; Uchimura et al., 2018). However, these bacterial metabolites or toxins may eventually contribute to disease or modulate health biochemically and immunologically without causing sepsis or infection, which is the so-called “leaky gut” (Obrenovich, 2018). Recently, a few studies have reported that gut microbiota can directly affect BBB development (Clarke et al., 2013). The gut can influence the BBB through gastrointestinal-derived hormonal secretion, allowing some drugs, amino acids, and small molecules to enter. For instance, a postmortem study found that 75% of individuals with autistic spectrum disorder (ASD) had reduced barrier-forming components while 66% showed increased expression of pore-forming molecules in duodenal tissues (Fiorentino et al., 2016). This suggests that the leaky gut and leaky brain may be linked. The gut microbiota represents a source of a significant amount of amyloids; although bacterial amyloids differ from CNS amyloids in their primary structure, they share similarities in their tertiary structure (Zhao et al., 2015). Therefore, bacterial amyloids may prime the immune system, consequently enhancing the brain’s neuronal amyloid production (Friedland and Chapman, 2017). In addition to bacterial amyloids, LPS produced by gut bacteria are also well-documented stimulators of neuroinflammation (Lee et al., 2008). LPS has been detected in hippocampal and neocortical brain lysates from AD patients, with most of it aggregating in the perinuclear region (Zhao et al., 2017). The leakage of bacterial amyloids and LPS from a leaky gut may directly contribute to the progression of AD.

### The Indirect Effects of Gut Microbes on AD

The microorganisms that colonize the intestine can help break down and metabolize ingested food, and some of the resulting metabolites, such as GABA, tryptophan, dopamine, and serotonin, may be involved in neurological functions (Tsavkelova et al., 2000; Shishov et al., 2009; Pokusaeva et al., 2017; Kaur et al., 2019). These neuroactive products generated in the intestine can reach the CNS *via* the bloodstream, where they act as neurotransmitters or their precursors or play other important roles (Briguglio et al., 2018). For instance, *Lactobacillus rhamnosus* JB-1 has been reported to produce GABA, which led to a reduction in depressive- and anxiety-like behaviors, with concomitant changes in cerebral GABAergic activity (Bravo et al., 2011). The composition of the intestinal microbial community changes constantly throughout the life cycle. Intestinal microbes promote the production of substances involved in signal transduction pathways and in regulating immune function by promoting the digestive ability of the host (Wang G. et al., 2019). However, the human body’s metabolism and immunity decline with age, which may lead to chronic inflammation, thereby increasing the risk of AD. In addition, in the case of malnutrition, the spread of opportunistic pathogens will lead to a significant decrease in the number of symbiotic microorganisms in the gastrointestinal tract, while a consequent decrease in the number of microorganisms with anti-inflammatory activity may also increase the risk of inflammation (Zheng et al., 2020). Evidence has shown that the intestinal microbiota can modulate the levels of circulating cytokines, which, in turn, has a significant impact on brain function (Forsythe and Bienenstock, 2010). For instance, several clostridial strains were shown to enhance T regulatory cell abundance and induce anti-inflammatory molecules, including IL-10, in mice (Atarashi et al., 2013), while proinflammatory molecules, including IL-1β and IL-6, were specifically associated with *Coprococcus comes* (Schirmer et al., 2016). The CNS and the enteric nervous system (ENS), which is the largest component of the peripheral nervous system, jointly regulate the function of the gastrointestinal tract. The pathogenic microorganisms that cause gastrointestinal diseases, such as gastric ulcers, may also act on the ENS and further affect the CNS. The two-way interaction between the gut microbes and the brain can be summarized as follows: microbial strains communicate through the vagus nerve connecting the brain and the digestive tract, and microbial-derived metabolites interact with the immune system to maintain communication between the microbes and the brain.

Sequencing of the 16S ribosomal RNA (rRNA) gene has facilitated the comparison of the gut microbiota composition among individuals, thereby revealing the correlation between specific microbes and disease. For instance, the depletion of the phyla *Firmicutes* and the increased abundance of *Proteobacteria* were linked to human IBD (Matsuoka and Kanai, 2015). On the other hand, butyrate, a short-chain fatty acid produced *by Ruminococcaceae, Eubacterium, Clostridia*, *and Firmicutes*, can exert anti-inflammatory effects in part by suppressing the activation of nuclear factor kappa-B (NF-κB) (Luhrs et al., 2002). The gut bacterial community structure differs significantly between AD model mice and their age-matched wild-type siblings. AD mice display significant reductions in the abundance of members of the phyla *Firmicutes*, *Verrucomicrobia*, *Proteobacteria*, and *Actinobacteria*, and increases in that of members of *Bacteroidetes* and *Tenericutes* (Harach et al., 2017); these changes may cause TNF-mediated inflammation of the gastrointestinal tract, thereby increasing the risk of AD (Schaubeck et al., 2016). This indicates that shaping the composition of the gut microbiota may influence the progression of AD. In 2019, Green Valley announced that sodium oligomannate (GV971), a marine-derived oligosaccharide reported to improve cognitive function in mild to moderate AD, had received conditional marketing approval in China. GV971 was reported to normalize the gut microbial profile and lessen brain immune cell infiltration and inflammation in AD mice (Wang X. et al., 2019), and was demonstrated to improve cognitive function in patients with mild-to-moderate AD as early as after 4 weeks of administration in a phase III trial. Mechanistically, GV971 was reported to reduce the levels of phenylalanine, which is involved in both the differentiation and proliferation of peripheral inflammatory TH1 cells; however, the link between phenylalanine and gut microbiota remains to be addressed (Wang X. et al., 2019).

LPS derived from gut microbiota has been shown to be more abundant in the AD brain and was found to be associated with Aβ plaques (Zhan et al., 2016; Zhao et al., 2017). LPS is a ligand for Toll-like receptor 4 (TLR4), which is highly expressed in brain microglia. The interaction between LPS and TLR4 activates the TLR4-mediated NF-κB and mitogen-activated protein kinase (MAPK) signaling pathways, thus triggering the release of proinflammatory cytokines, resulting in neurodegeneration and abnormal cognitive behavior (Huffman et al., 2019). This may support the hypothesis that an increased abundance of LPS-producing bacteria can activate immune cells and enhance neuroinflammation, and further suggests that targeting the gut microbiota may be a putative therapy for the treatment of AD.

## The Relationship Between Oral and Intestinal Microbiota

Because oral microorganisms and the intestinal microbiota are different, especially in terms of bacteria, the oral microorganisms that enter the gastrointestinal tract with saliva may, to a certain extent, change the structure of the intestinal microbial community, leading to metabolic endotoxins, which will further induce inflammation-related changes in various tissues and organs. For example, *P. gingivalis* entering the intestine through swallowing will change the composition of the intestinal microbiome and further increase the permeability of the intestinal epithelium (Arimatsu et al., 2014; Feng et al., 2020). Other studies have shown that the administration of *P. gingivalis* can cause changes in the intestinal microbiota, and even induce the upregulation of the mRNA expression of various proinflammatory cytokines (Kato et al., 2018; Ohtsu et al., 2019). A leaky gut has been reported to be associated with older adults; therefore, both *P. gingivalis* that survive in the gut and gut-resident microbiota or their metabolites may pass into the bloodstream of the ENS and enter the brain. These data support that periodontal disease induces a gut-mediated systemic pathology.

## Conclusion

**FIGURE 1:**
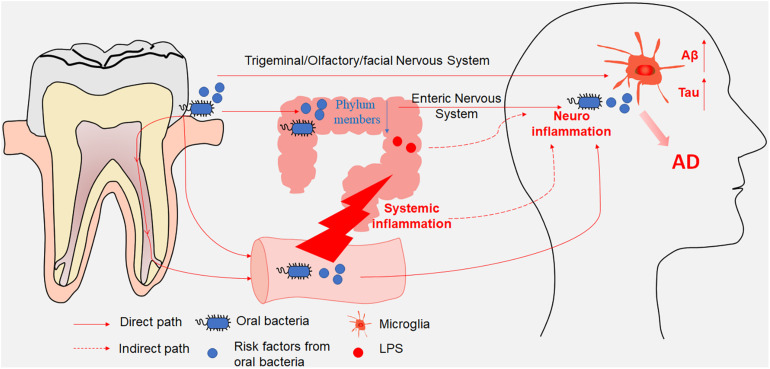
A schematic illustration depicting the paths through which oral and intestinal microbiota enter the central nervous system (CNS). Oral bacterial species and their products affected the brain either directly through the trigeminal/olfactory/facial nervous system and circulating blood or indirectly through gut microbiota disturbance. Meanwhile, gut microbiota and their products affected the brain either directly through the enteric nervous system or indirectly through mediating systemic inflammation. Both direct and indirect effects from oral and intestinal microbiota contributed to the microglia-mediated neuroinflammation, resulting in AD-related pathologies.

Oral bacterial species and their products can affect the brain either directly through the trigeminal/olfactory/facial nervous system and circulating blood or indirectly through gut microbiota dysbiosis and systemic inflammation (Table 1). The gut microbiota and their products can affect the brain either directly through the ENS or indirectly through mediating systemic inflammation. Both direct and indirect effects from oral and intestinal microbiota can contribute to microglia-mediated neuroinflammation, resulting in AD-related pathologies (Figure 1). Consequently, oral- and intestinal-specific bacterial species and their products may be potential biomarkers for the prevention and clinical diagnosis of AD. Current research has shown that chronic neuroinflammation caused by certain oral or intestinal microbes occurs several years or even decades before the emergence of cognitive impairment. The timely use of antibiotics to treat periodontitis or probiotics and prebiotics to treat gut dysbiosis may prevent cognitive impairment and AD. One study in mice showed that broad-spectrum antibiotics did not protect against *P. gingivalis*-induced cell death due to the rapid acquisition of resistance by the bacterium, whereas a gingipain inhibitor did (Dominy et al., 2019). An ongoing clinical trial of the gingipain inhibitor (ClinicalTrials.gov NCT03331900) may further confirm the results of the animal studies. Meanwhile, an antibiotic cocktail (ABX)-mediated perturbation of the gut microbiota was associated with reduced Aβ plaque pathology and astrogliosis in male AD mice (Dodiya et al., 2019); in contrast, an observational cohort study found that patients in whom antibiotic treatment was withheld had more severe dementia compared with those who received antibiotics with curative intent (Van Der Steen et al., 2002). These contradictory results between model animals and human patients may on account of the patient age, aspiration, and history of pneumonia. Based on increasing evidence from both mouse models and clinical studies indicating that the composition of the oral and intestinal microbiota can influence cognitive function, future studies should identify (1) the source of the microorganisms detected in the brains of AD patients; (2) the specific pathways by which specific microbiota and their metabolites enter the brain parenchyma; (3) whether early periodontal disease treatment can improve cognitive function in clinical studies; and (4) the mechanisms used by the microbiota to evade the immune system in the circulation and the CNS.

**TABLE 1:** Microorganisms associated with AD.

Microorganism name	Main colonization	References
*Candida albicans*	Oral cavity, Intestine	Bulgart et al., 2020
*Escherichia coli*	Intestine	Zhan et al., 2016; Zhao et al., 2017; Bulgart et al., 2020
*Staphylococcus epidermidis*	Intestine	Bulgart et al., 2020
*Staphylococcus aureus*	Intestine	Bulgart et al., 2020
*Listeria monocytogenes*	Intestine	Bulgart et al., 2020
*Enterococcus faecalis*	Intestine	Bulgart et al., 2020
*Pseudomonas aeruginosa*	Intestine	Bulgart et al., 2020
*Streptococcus mitis*	Oral cavity	Bulgart et al., 2020
*Streptococcus salivarius*	Oral cavity	Bulgart et al., 2020
*Porphyromonas gingivalis*	Oral cavity	Liu et al., 2013; Dominy et al., 2019; Ohtsu et al., 2019; Gu et al., 2020; Zeng et al., 2020
*Helicobacter pylori*	Intestine	Zhan et al., 2016
*herpesviruses*	Oral cavity	Zhan et al., 2016
*cytomegalovirus*	Oral cavity	Zhan et al., 2016
*Bacteroides fragilis*	Intestine	Zhao et al., 2017

## Author Contributions

N and JN conceived and drafted the manuscript. All other authors made significant contribution and approved the manuscript. All authors contributed to the article and approved the submitted version.

## Conflict of Interest

The authors declare that the research was conducted in the absence of any commercial or financial relationships that could be construed as a potential conflict of interest.
